# Effect of Carbon Black Nanofiller on Adhesion Properties of SBS Rubber Surfaces Treated by Low-Pressure Plasma

**DOI:** 10.3390/polym12030616

**Published:** 2020-03-08

**Authors:** Jacek Tyczkowski, Jacek Balcerzak, Jan Sielski, Iwona Krawczyk-Kłys

**Affiliations:** 1 Department of Molecular Engineering, Faculty of Process and Environmental Engineering, Lodz University of Technology, Wolczanska 213, 90-924 Lodz, Poland; jacek.balcerzak@p.lodz.pl (J.B.); jan.sielski@p.lodz.pl (J.S.); 2 Institute of Leather Industry, Zgierska 73, 91-462 Lodz, Poland; i.krawczyk-klys@ips.lodz.pl

**Keywords:** SBS rubber, nano-carbon black, cold plasma, polymer surface modification, adhesion, bound rubber

## Abstract

Studies on the surface modification of commercial styrene-butadiene-styrene (SBS) rubber with different carbon black (CB) nanofiller content (10–80 parts per hundred parts of rubber (phr)) performed by low-pressure oxygen plasma are presented in this paper. The adhesion properties of the rubber were determined by the peel test for adhesive-bonded joints prepared with a water-based polyurethane (PU) adhesive. The chemical structure and morphology of the SBS rubber surface before and after plasma treatment were investigated by X-ray photoelectron spectroscopy (XPS) and scanning electron microscopy (SEM), respectively. The peel tests showed that the plasma treatment significantly improved the strength of adhesive-bonded joints in the entire range of CB tested, revealing a clear maximum for approximately 50 phr of CB. It was also found that as a result of plasma treatment, functional groups that are responsible for the reactions with the PU adhesive, such as C−OH and C=O, were formed, and their concentration, similar to the peel strength, showed maximum values for approximately 50 phr CB. The occurrence of these maxima was explained using the bound rubber model.

## 1. Introduction

Adhesive bonding of rubbers to other materials, such as artificial leather, textiles, plastics, etc., is a particularly important issue in various production processes, for example in the footwear and automotive industries. It is obvious that the strength and quality of such adhesive-bonded joints depend, to a large extent, on the chemical structure and morphology of the rubber surface and, therefore, can be controlled by various surface treatments. One of the typical methods to improve the adhesion of rubbers (based on SBS copolymers) to polyurethane (PU) adhesives is the wet chemical chlorination of the rubber surface [1,2]. This method, however, has serious disadvantages arising from the fact that very toxic and hazardous substances are released to the environment. Thus, it is no wonder that we are looking for cleaner, more energy-efficient and environmentally friendly alternatives to the wet chemical method. Cold (non-equilibrium) plasma treatment has proven to be a particularly useful method to meet these expectations [3,4,5,6,7,8,9,10].

Considering the potential and benefits of the surface treatment by cold plasma, many studies have been carried out using this method to improve the joints between SBS elastomers and PU adhesive. In general, a drastic increase in the adhesion of the elastomer surface to the adhesive was observed after such a treatment. In some cases, only a few seconds of plasma exposure was enough to obtain several times higher peel strength than that for the non-treated samples. The plasma treatment, which can be performed both in inert plasmas (e. g. Ar, He) and chemically reactive plasmas (e. g. O_2_, CO_2_, H_2_O), consists in changing the chemical structure of the elastomer surface by bombarding it with ions, which leads to the preferential etching of the surface by removing certain atoms or their groups, as well as creating a large number of radical centers. On these centers, some functional groups, such as hydroxyl (−OH), carbonyl (>C=O), etc., can be formed directly in the plasma processes (reactive plasmas) or further after the contact of the surface with air atmosphere (inert plasmas). The resulting functional groups can react with diisocyanates, which are typical prepolymer components of PU adhesives. The isocyanate group on one side of the diisocyanate molecule can form a chemical bond to the SBS elastomer surface, while the other group on the opposite side (through reactions with other components (e.g. polyols) or PU oligomers, form a final polymeric structure of the adhesive [11,12]. The concentration of functional groups, which depends on the plasma process conditions, is one of the main factors responsible for the strength of SBS−PU joints [9,13].

So far, most of the research carried out in the area of plasma surface treatment of SBS rubbers has been limited only to SBS elastomer models, dealing with commercial rubbers only occasionally [5,14,15]. Regardless of the fact that the elastomer consists of different polymer blocks (polystyrene and polybutadiene) that interact differently with plasma [4,16], the produced rubbers are blended with ingredients such as carbon black, silica, zinc oxide, etc. We are still far from a thorough knowledge of changes in the molecular structure of the rubber surface caused by interaction with the plasma, and consequently, the relationship between the plasma treatment and the adhesive strength of the rubber surface. Research in this area is therefore fully justified, especially considering the application point of view.

One of the important ingredients added to the rubber, having a significant impact on its properties, is carbon black (CB) in the form of nanoscale particles. Indeed, the rubber industry has used this modification extensively to improve abrasion resistance, elastic modulus, tensile strength, viscoelasticity as well as rheological and conductive properties of elastomeric composites [17,18,19,20,21,22]. However, despite decades of such technology, the actual mechanisms by which CB nanoparticles modify the macroscale properties of rubbers are still not fully understood [23]. Nevertheless, there is no doubt that an important role in these phenomena is played by the molecular interaction between the filler nanoparticles and polymer chains. The concept of bound rubber at the particle−polymer interface has been put forth to describe such an interaction [23,24,25]. The bound rubber is a volume of the polymer fraction directly adjacent to the interface, having a molecular structure that differs from that in the pure polymer—an interphase layer with a thickness lying in the range of 2−80 nm is formed. The total volume of this fraction depends on the concentration, shape, and size of the filler nanoparticles, and also their primary aggregation and clustering [26,27,28,29].

The formation of the bound rubber with a different structure than that of the pure polymer creates the suspicion that these two types of material behave differently under the effect of plasma treatment. It could be manifested in the influence of the amount of CB added to the rubber on its surface properties after such a treatment. This work aims to investigate the effect of CB contained in the SBS rubber on the adhesive strength of its surface treated with cold low-pressure oxygen plasma.

## 2. Materials and Methods 

### 2.1. Materials

The study was carried out on a commonly used vulcanized commercial rubber based on styrene-butadiene block copolymer (SBS). The rubber was prepared by “Kwarciak PPHU” Company (Kłomnice, Poland). The detailed composition of the rubber is presented in Table 1. Only the loading of CB (N330) was changed from 10 to 80 phr, leaving the contents of all other components at the same level. The carbon black N330 (Carbex 330, produced by the Car-bochen, Gliwice, Poland) is a technical furnace black with a mean size of particles of 30 nm, a specific surface of 80−100 m^2^/g, and a bulk density of 350 g/dm^3^. KER 1502 (SBS) with 76.5 wt. % of butadiene content (chemically bounded) was used as the main copolymer of the rubber compound. Some amount of KER N-29 (nitrile−butadiene copolymer) with 71 wt. % of butadiene (chemically bounded) was added to the rubber compound. (Both copolymers were supplied by Synthos Dwory Ltd., Oświęcim, Poland.) The rubber preparation process consisted of two stages. First, all components were mixed using a rubber blender (mixing time was 8 minutes, the speed was 32 rpm and the temperature was 120 °C). Then the rubber compound was machined in a “ZGODA 550 L” rolling mill (ZGODA, Świętochłowice, Poland) with a diameter of 550 mm and length of 1500 mm, at a regulated temperature of 60 °C at 20 rpm and roller friction 1:1.25. The obtained product was stored at room temperature without light.

### 2.2. Plasma Treatment

The plasma treatment was carried out in a parallel plate reactor with a radio frequency (RF, 13.56 MHz) glow discharge. A detailed description of the reactor is given in [30]. Plasma was generated in a reactor chamber containing oxygen (pure O_2_, Air Liquide, Cracow, Poland) with a flow rate of 7.5 sccm and an initial pressure of 13 Pa. The power of the glow discharge was 50 and 80 W and the plasma treatment of the rubber samples lasted 2 min. These parameters were selected from the group of the most suitable plasma treatment parameters for the SBS rubber to improve its adhesive capacity to PU adhesives, which was determined based on a series of tests carried out under the R&D project that is mentioned in the Acknowledgments.

### 2.3. Peel Tests

To determine the adhesive strength of the rubber surfaces, 180°-peel tests, according to the European Standard EN 1392:2007, were carried out. Adhesive-bonded joints were prepared using the rubber samples before and after the plasma treatment (originally with a mechanically roughened surface) and strips of standard leather (boxcow, chrome-tanned, non-pigmented). Two-component, water-based PU adhesive (Bonidur Us-100 + 5.0 wt. % of curing agent Bopherem I-10; supplied by Bochem Ltd., Pionki, Poland) was spread on each adherend and dried at room temperature for 30 min. The dry adhesive films were activated by heating at 353 K for 3 min and the surfaces were immediately contacted under a pressure of 0.4 MPa for 15 s. The adhesive joints were then conditioned for 48 h at room temperature. The peel strength measurements were performed using a tensile tester model 5566 (Instron, High Wycombe, UK) at a peel rate = 1.67 × 10^−3^ m/s. The average value of the peel strength for a given type of surface was determined from at least three samples and a minimum of 10 measurement points for each of them.

### 2.4. X-ray Photoelectron Spectroscopy (XPS)

Surface chemical characterization of the SBS rubber samples to a depth of several nm was carried out using AXIS Ultra photoelectron spectrometer (XPS, Kratos Analytical Ltd., Manchester, UK) equipped with a monochromatic Al−Kα X-ray source (1486.6 eV). The power of the anode was set at 150 W, and the hemispherical electron energy analyzer was operated at pass energy 20 eV for all high-resolution measurements. All measurements were carried out with a charge neutralizer. The component of C1s line, assigned to C−C / C−H / C=C, and positioned at 284.8 eV, was used to calibrate the spectra.

### 2.5. Scanning Electron Microscopy (SEM)

A scanning electron microscope Quanta 200 F (FEI, Hillsboro, OR, USA) equipped with a Large Field Detector (LFD) was used to study the surface topography of SBS rubbers. All measurements were carried out under a nitrogen atmosphere of 100 Pa. The topography was analyzed using electron energy of 3.5 keV, which resulted in penetration depth of approximately 100 nm, as it was estimated based on Ref. [31]. 

## 3. Results and Discussion

**Figure 1:**
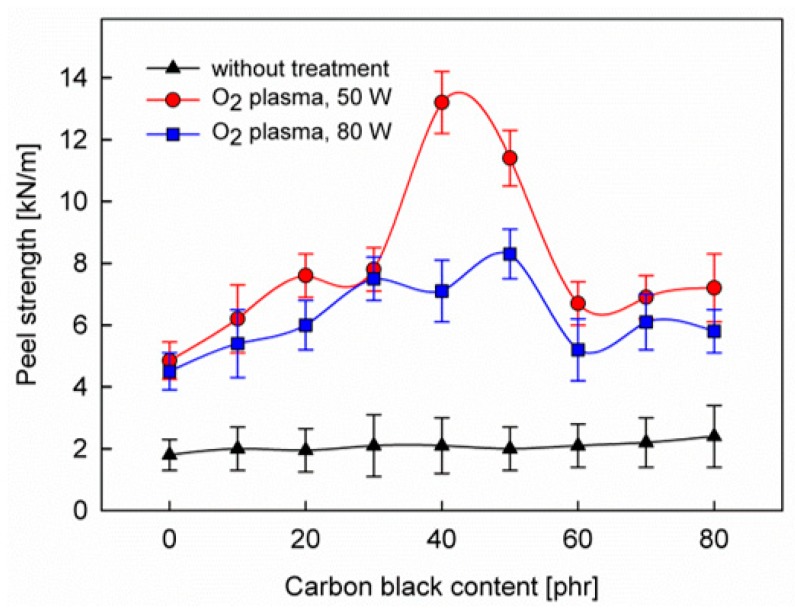
Peel strength test for the SBS rubber with various carbon black content, before and after plasma treatments.

The most important feature of the rubber surfaces, from their application point of view, is the strength of adhesive-bonded joints formed between such surfaces and other materials. Therefore, the fundamental attention in the work was focused on the peel strength test which, on the one hand, was to show the effect of CB contained in the rubber on the joint strength, and, on the other hand, to determine the role of plasma treatment in the formation of this joint. Results of the peel strength test performed for untreated as well as plasma-treated SBS-rubber samples with various amounts of the CB are shown in Figure 1. As can be seen, the plasma treatments, irrespective of the discharge power used, produce a considerable improvement in the adhesion properties of the rubber surface in the entire range of CB tested. Peel strength values for the untreated samples are significantly lower. This result is not surprising, as many previous studies have shown a significant increase in the strength of joints formed by rubbers and elastomers after the plasma treatment of their surfaces [4,7,9,15,30].

Also understandable is the higher peel strength of the adhesive joint after plasma treatment with the discharge power of 50 than 80 W. This is related to the competition between the crosslinking process and the formation of functional groups responsible for the adhesive bonding process (mainly −OH groups). At higher discharge powers, the crosslinking process begins to dominate and the concentration of these functional groups decreases. This problem has already been described in detail elsewhere [4,9]. Another problem that should be considered is the nano-roughening of the rubber surface under plasma treatment and its participation in the adhesive-bonded joints in the form of mechanical adhesion [9]. In our case, however, under the used plasma parameters and plasma treatment times as short as 2 min, the effect of the nano-roughness generated by plasma treatment on the joint strength can be negligible compared to the process of chemical bond formation. This is supported by investigations performed by AFM microscopy for plasma-treated SBS surfaces [32]. The general fact that nano-roughness formation is more intensive for higher discharge power [33] also does not confirm in our case the contribution of this effect to the adhesive bonding process—as shown in Figure 1, the peel strength for 50 W is greater than for 80 W.

What is most interesting, however, is that the dependencies of the peel strength on the CB content for the plasma-treatment samples show maximum values. With the increase in the CB content, the peel strength of the adhesive joint initially increases, and then, from approximately 50 phr, begins to decrease. This is in contrast to the dependence for untreated samples, where the peel strength is practically independent of the CB content.

**Figure 2:**
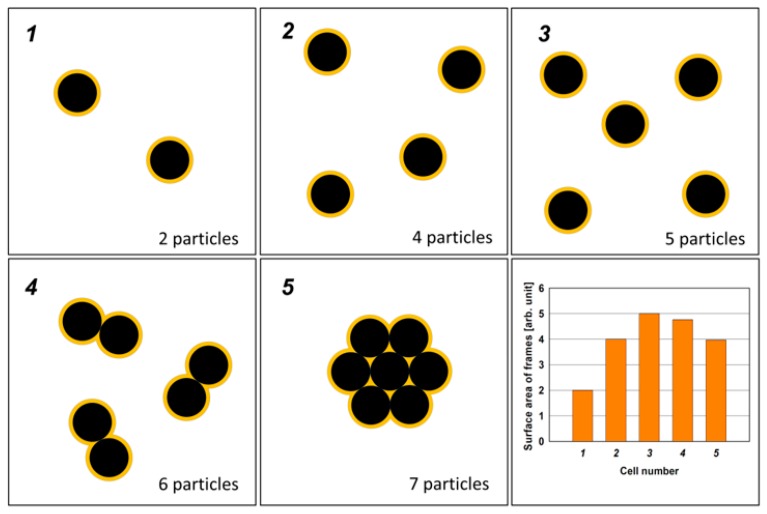
A simple model of the rubber surface with carbon black nanoparticles surrounded by bound rubber (yellow frames). As the carbon black content increases, the clustering effect occurs. The graph (bottom right) shows the changes in the surface area occupied by the bound rubber as a function of the carbon black content in this model. (The graph was obtained using the GIMP 2.10.18 software [34], by which the number of pixels for the surface area of nanoparticles and bound rubber was counted in the presented model.)

To explain the maxima in Figure 1, the concept of bound rubber seems to be particularly useful. The CB particles are coated by a very thin interphase layer created from polymer chains with a subtle molecular structure that differs from that in the pure polymer. Looking at the surface of the rubber, we see particles of CB (primary aggregations) in the form of uncovered carbon nano-islands surrounded by narrow zones (with a width of nanometers) of the bound rubber (Figure 2). Increasing the content of the CB, we increase the number of its particles, so the number of islands and the total area occupied by the bound rubber also increases. However, by increasing the content of CB, we also increase the likelihood of clustering of its particles. The islands begin to become larger by clustering, and consequently, the surface area of the bound rubber surrounding the islands begins to decrease (see: the bar chart in Figure 2). Thus, the dependence of the surface area occupied by the bound rubber on the volume fraction of CB passes through the maximum [28].

Looking for an analogy between the maximum that occurs in the surface area occupied by the bound rubber and the maximum in the peel strength for the increasing content of CB, it should be assumed that the structure of the bound rubber is much more susceptible to the formation of functional groups (−OH, >C=O) by plasma treatment compared to the rest of the polymer. Indeed, investigations performed by Choi [26] suggest that the butadiene units of SBS elastomer are more compatible with the CB than the styrene units. Thus, since the butadiene units are attached to the CB particles more readily than the styrene units, the ratio of the butadiene/styrene components is higher in the bound rubber region than in the compounded rubber. Choi and Kim [27] have also shown that the bound rubber in the case of SBS rubber has more butadiene units with 1,2-configuration than the raw polymer because the 1,2-units interact more strongly with the CB particles than the butadiene units with 1,4-*cis* and 1,4-*trans* configurations. On the other hand, our previous studies [4] have shown that much more susceptible to the formation of −OH and >C=O functional groups by means of plasma treatment are butadiene units than the styrene units, and in turn, among the butadiene units, those with 1,2-configuration are more active than 1,4-*cis* and 1,4-*trans*.

In summary, it can be concluded that the increase of CB content to a certain value increases the surface area occupied by the bound rubber, which in the case of SBS rubber is much richer in butadiene units with 1,2-configuration compared to the rest of the polymer. Accordingly, the concentration of plasma-generated functional groups that are responsible for the reactions with the PU adhesive increases in this range of CB content, and as a consequence, the peel strength of the adhesive joint is increased. For CB content greater than 50 phr, the surface occupied by the bound rubber decreases (due to the clustering of its particles), so the joint strength decreases.

**Figure 3:**
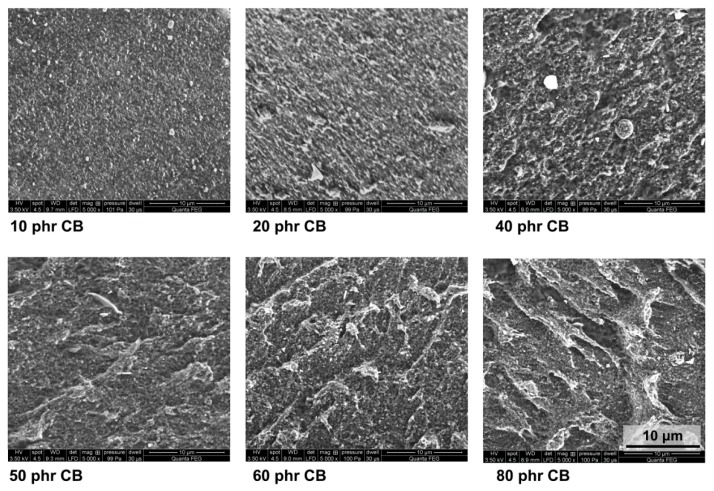
SEM images of the SBS rubber with different carbon black (CB) content. All the images with the same magnification (5000×).

The clustering process that underlies the observed dependence of the peel strength of the adhesive joint on the CB content (Figure 1) is illustrated by electron microscopy studies. SEM images of the SBS rubber with different CB content are shown in Figure 3. The evident differences in surface morphology (that was sampled to a depth of approximately 100 nm) seen in the subsequent images should be attributed to the increase in CB content and the formation of increasingly large clusters. We assigned brighter spots in the SEM images to the CB aggregates and clusters. This conclusion is based on the SEM procedure used for imaging, in which the images were recorded with the LFD detector.

The LFD detector captures the secondary electrons, but also the backscattered electrons that carry information on the atomic number contrast. It has been shown that the backscattered electron contrast of a mixture of atoms that is homogeneous on the atomic scale can be accurately predicted from the mass concentrations of the elemental constituents. The measured signal follows a predictable response to a specimen property of interest, such as composition. Thus, the regions with higher mass concentrations (CB) should be seen as brighter spots compared to those of lower mass concentrations (polymer). However, it should be noted that the dimension of these spots is generally larger than the real size of the objects due to the scattering process [31].

**Figure 4:**
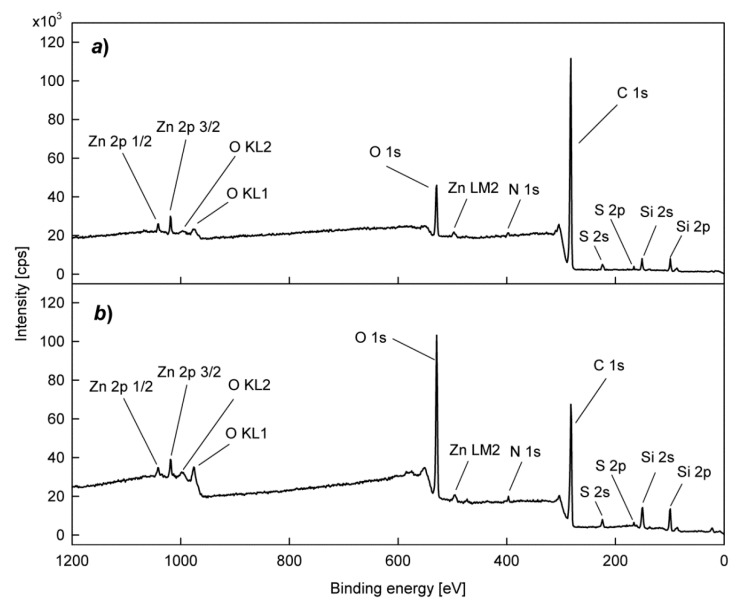
XPS wide scan spectra for SBS rubber with 50 phr of carbon black: (**a**) before plasma treatment; (**b**) after plasma treatment (O_2_ plasma, 80 W).

**Figure 5:**
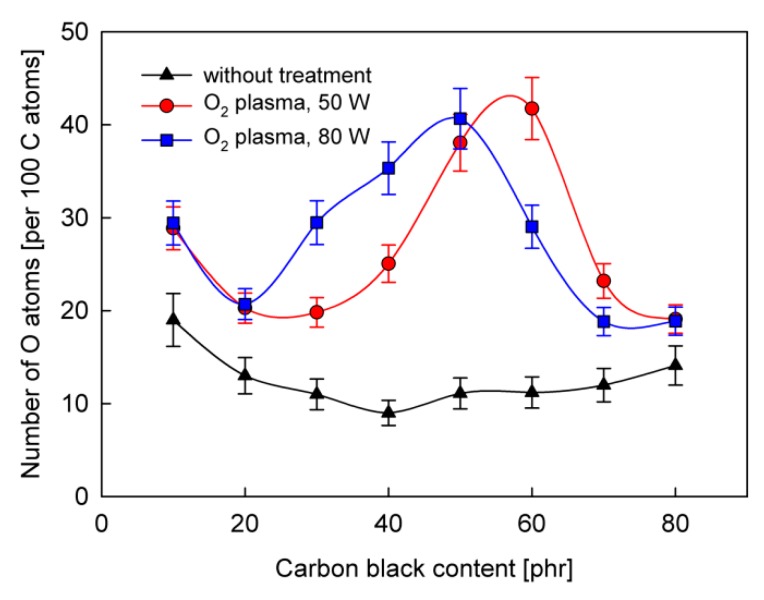
The concentration of oxygen atoms on the surface of SBS rubber with various carbon black content, before and after plasma treatments.

To confirm the predicted relationship between the CB content and the concentration of functional groups generated by plasma treatment, XPS studies were performed on the chemical structure of the surface of SBS rubber samples. Typical XPS wide scan spectra for an SBS rubber sample before and after plasma treatment are presented in Figure 4. In addition to the main carbon (C1s) and oxygen (O1s) bands, we also see weak bands for zinc and silicon and trace bands for nitrogen and sulfur, which is compatible with the rubber composition. The plasma treatment, as shown in Figure 4, primarily causes a significant increase in the number of oxygen atoms in relation to the number of carbon atoms. Changes in the oxygen concentration determined in this way on the rubber surface, both before and after plasma treatment, depending on the CB content, are illustrated in Figure 5. While the untreated samples show low values of oxygen concentration, after the plasma treatment we can see much higher values with clearly visible maxima for about 50 phr of CB. The increase in the surface concentration of oxygen should be attributed to the formation of oxygen functional groups.

**Figure 6:**
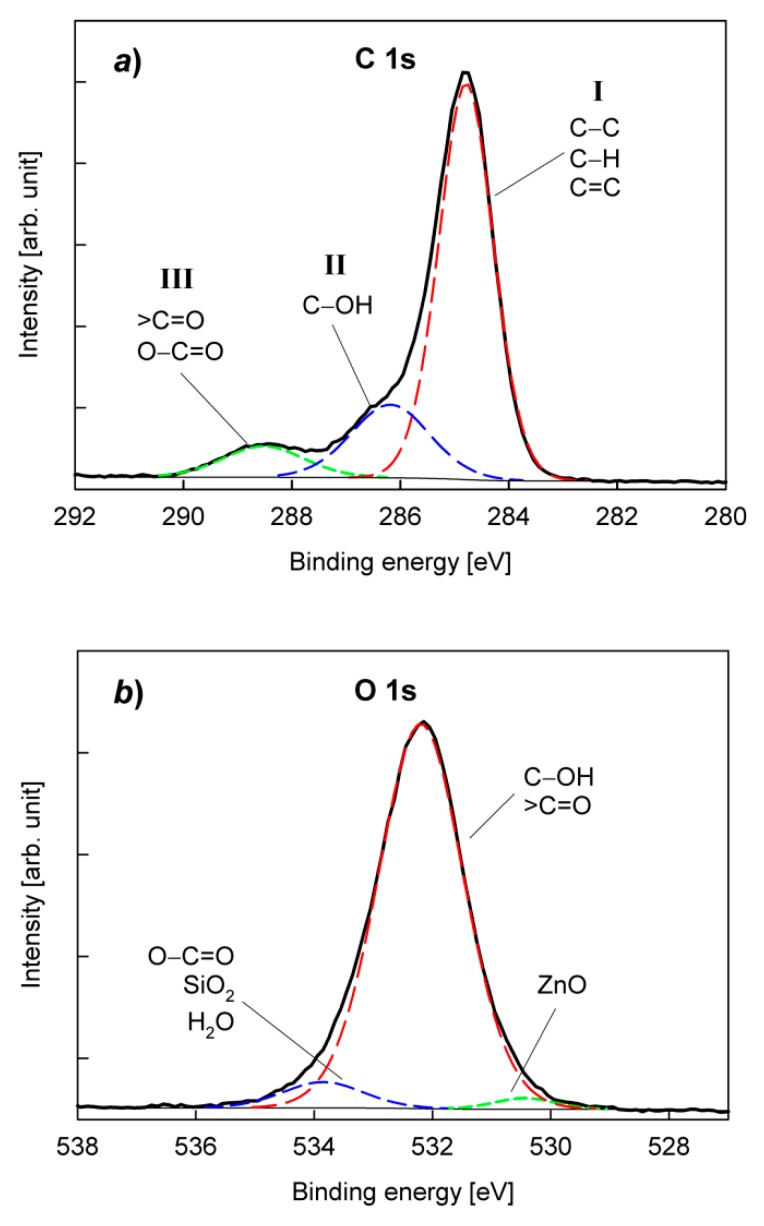
XPS narrow spectra for SBS rubber with 50 phr of carbon black, after plasma treatment (O_2_ plasma, 50 W): (**a**) C1s; (**b**) O1s.

Considering the chemical structure of functional groups involved in the formation of bonds with the PU adhesive, which are composed of carbon and oxygen atoms, two regions in the XPS spectra, namely for C1s and O1s, were analyzed in detail. Examples of C1s and O1s spectra are shown in Figure 6.

The spectra were numerically deconvoluted and the obtained bands were assigned to appropriate species, according to our previous paper and references therein [30]. As is shown in Figure 6a, the C1s spectrum can be fitted by three bands. The band I fixed at 284.8 eV corresponds to C−C, C=C and C−H units. The band II (at 286.0−286.4 eV) is usually attributed to C−OH. In turn, the band III (at 288.0−288.8 eV) can be assigned to >C=O and O−C=O groups. The analysis of the O1s spectrum is, unfortunately, more complicated. Roughly, the spectrum O1s can also be fitted with three component bands (Figure 6b). The main band at 532.0−532.5 eV is associated with C−OH and >C=O groups. However, attempts to split this band into two bands corresponding to these two groups do not give satisfactory results. There is also a problem with the band at 533.8−534.1 eV, which can be attributed to the O−C=O group. Besides, an O1s band for SiO_2_ should also be expected in the same position. H_2_O molecules strongly attached to SBS rubber via hydrogen bonds can also give a band in this region. The third band (at 529.9−530.4) is most likely related to O1s for ZnO.

**Figure 7:**
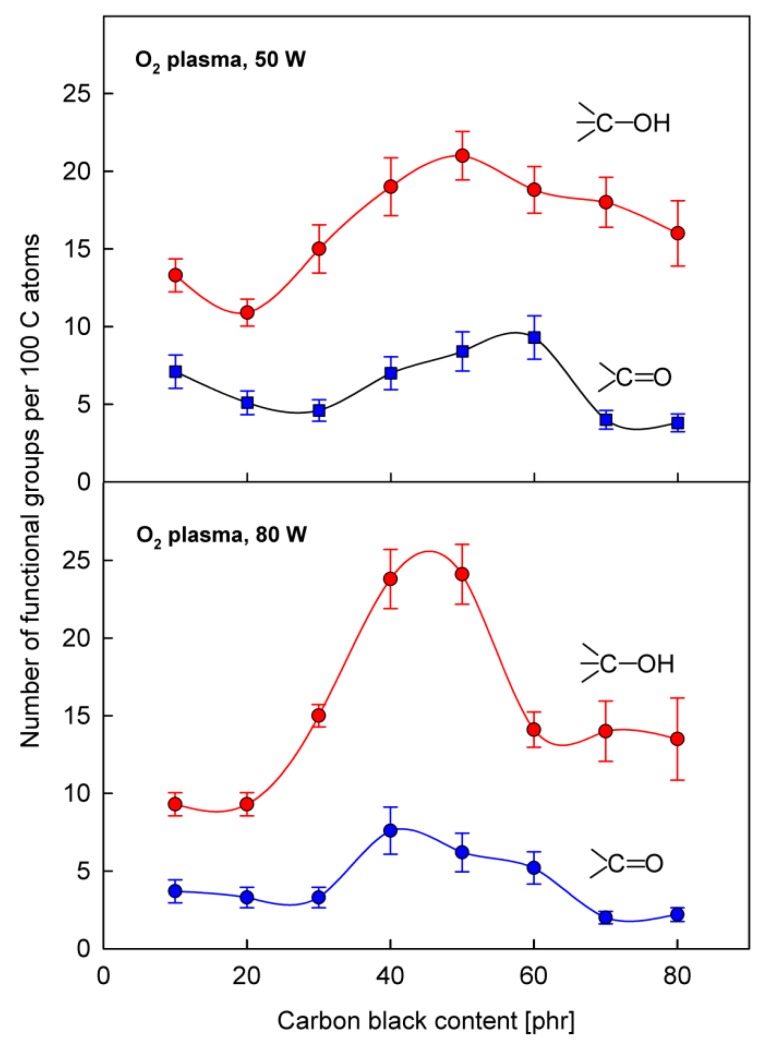
The concentration of functional groups (C−OH, >C=O) on the surface of SBS rubber with various carbon black content, after plasma treatments.

As one can see, the complicated structure of the O1s spectrum makes it impossible to carry out a detailed analysis of the concentration of C−OH and >C=O functional groups on the SBS rubber surface. This analysis was therefore carried out based on the C1s spectrum. Figure 7 shows the C−OH and >C=O concentrations calculated roughly on the bases on bands II and III, respectively, as a function of the CB content for two different plasma powers. The pronounced maxima that appear in these relationships in the range around 50 phr of CB are in good coincidence with the maximum of the peel strength occurring in the same range (Figure 1). This result, firstly, confirms a correlation between changes in the concentration of C−OH and >C=O function groups and changes in the strength of the joint bonded using PU adhesive and, secondly, it shows that the formation of these groups as a result of plasma treatment is most likely, to a large extent, associated with the bound rubber, the area of which on the rubber surface also passes through the maximum during the increase of the CB content, as discussed above (Figure 2). It should be emphasized that the area of the bound rubber is the only part of the surface area, the value of which passes through the maximum with the increase in the CB content; at the same time, the CB area is constantly growing, while the SBS elastomer area is decreasing.

The results shown in Figure 7 should, however, be treated more qualitatively than quantitatively, because the total number of C−OH and >C=O groups consists of groups formed at different places on the rubber surface (i.e. SBS blocks, CB particles, the other ingredients appearing on the surface, and especially important from our point of view—the bonded rubber zones), which entails different interactions with the adhesive. In turn, the concentrations of these different oxygen group fractions are closely related to the plasma treatment parameters (e.g. discharge power). Therefore, in such a complex system as the tested rubber, it is difficult to expect a simple linear correlation between the concentration of groups and the joint strength, as it was observed in the model SBS elastomer [13]. Nevertheless, there is no doubt that the dependencies in Figure 7 reveal the maxima, which justifies the proposed explanation of the influence of CB content on the adhesive properties of the SBS rubber tested with the PU adhesive.

## 4. Conclusions

Apart from the statement that the treatment of SBS rubbers by O_2_ plasma drastically improves the strength of adhesive-bonded joints between the rubber surface and the water-based PU adhesive, the investigations performed within the scope of this work bring us closer to understanding the plasma processes taking place on the surface of commercial SBS rubbers, and primarily, to determine the effect of the CB content added to the rubber as a nanofiller on these processes. It has been found, which is particularly interesting, that with the increase of the CB content, the peel strength of the adhesive joint initially increases, and then, from approximately 50 phr of CB, it begins to decrease. A very similar dependence has also been established for changes in the concentration of functional groups (C−OH, >C=O) formed by the plasma treatment, which are responsible for reactions with the PU adhesive. The pronounced maxima are visible in the range around 50 phr of CB, which is in good coincidence with the maximum of the peel strength occurring in the same range. This confirms the assumption that the chemical nature of the adhesion is a key factor governing the adhesive bonding process after the SBS rubber treatment by O_2_ plasma. Besides, it also suggests that the formation of the functional groups is closely related to the bound rubber that forms very narrow zones around the CB particles on the rubber surface. Indeed, the chemical structure of this material in the case of SBS polymer seems to be particularly susceptible to plasma treatment. With the increase of the CB content, the area occupied by the bound rubber also increases initially, and then due to clustering of the CB particles, passes through the maximum, which may explain the dependence of the concentration of the functional groups and the adhesive joint strength on the CB content.

Although the above interpretation based on the concept of bound rubber seems to be reasonable, it requires further research, relevant not only on the basic level but also for industrial applications.

## Figures and Tables

**Table 1:** The compound composition of the SBS rubber.

Ingredient	Content [phr] ^a^
Styrene-butadiene (SBS) copolymer (KER 1502)	100
Nitrile-butadiene copolymer (KER N-29)	42.9
Carbon black (N330)	10 to 80
Silica (Arsil)	11.5
Zinc oxide	5.7
Phthalates	5.7
Tetramethyl thiuram disulfide	2.3
Oiled sulfur	1.7
*N*-cyclohexyl-2-benzothiazole sulfenamide	1.7
Antioxidant	1.4
Protector G35	1.1
Stearin	1.0

^a^ phr = parts per one hundred parts of SBS copolymer.

## References

[1] Martin-Martinez J.M., Bhowmick A.K. (2008). Improving adhesion of rubber. Current Topics in Elastomers Research.

[2] Radabutra S., Thanawan S., Amornsakchai T. (2009). Chlorination and characterization of natural rubber and its adhesion to nitrile rubber. Eur. Polym. J..

[3] Ortiz-Magán A.B., Pastor-Blas M.M., Martin-Martinez J.M., d’Agostino R., Favia P., Oehr C., Wertheimer M.E. (2005). Different performance of Ar, O_2_ and CO_2_ RF plasmas in the adhesion of thermoplastic rubber to polyurethane adhesive. Plasma Processes and Polymers.

[4] Tyczkowski J., Krawczyk I., Woźniak B., d’Agostino R., Favia P., Oehr C., Wertheimer M.E. (2005). Plasma−surface modification of styrene-butadiene elastomers for improved adhesion. Plasma Processes and Polymers.

[5] Romero-Sánchez M.D., Pastor-Blas M.M., Martin-Martinez J.M. (2005). Environmental friendly surface treatments of styrene-butadiene-styrene rubber: alternatives to the solvent-based halogenation treatment. Int. J. Adhes. Adhes..

[6] Cognard J. (2006). Some recent progress in adhesion technology and science. Compt. Rend. Chim..

[7] Anagreh N., Dorn L., Bilke-Krause C. (2008). Low-pressure plasma pretreatment of polyphenylene sulfide (PPS) surfaces for adhesive bonding. Int. J. Adhes. Adhes..

[8] Gao S.H., Zhou K.S., Lei M.K., Wen L.S. (2008). Surface modification of silicone rubber by CF_4_ radio frequency plasma immersion. Plasma Chem. Plasma Proc..

[9] Tyczkowski J., Makowski P., Krawczyk-Kłys I., Wójcik J. (2012). Surface modification of SBS rubber by low-pressure inert gas plasma for enhanced adhesion to polyurethane adhesive. J. Adhes. Sci. Technol..

[10] Henry A., Vallat M.F., Noël C., Belmonte T., Roucoules V. (2015). Influence of plasma chamber set-up on the surface modification of non-vulcanized and pure SBR rubber treated at radio-frequencies air plasma. Plasma Proc. Polym..

[11] Petrie E.M. (2000). Handbook of Adhesives and Sealants.

[12] Segura D.M., Nurse A.D., McCourt A., Phelps R., Segura A., Cognard P. (2005). Chemistry of polyurethane adhesives and sealants. Adhesives and Sealants.

[13] Tyczkowski J., Krawczyk-Kłys I., Kuberski S., Makowski P. (2010). Chemical nature of adhesion: Plasma modified styrene–butadiene elastomer and polyurethane adhesive joints. Eur. Polym. J..

[14] Krawczyk-Kłys I., Makowski P., Wójcik J., Tyczkowski J. (2012). Plasma surface modification of commercial SBS rubbers for enhanced adhesive bonding. Mater. Sci-Medzg..

[15] Cantos-Delegido B., Martin-Martinez J.M. (2015). Treatment with Ar−O_2_ low-pressure plasma of vulcanized rubber sole containing noticeable amount of processing oils for improving adhesion to upper in shoe industry. J. Adhes. Sci. Technol..

[16] Tyczkowski J., Krawczyk I., Woźniak B. (2003). Modification of styrene−butadiene rubber surfaces by plasma chlorination. Surf. Coat. Technol..

[17] Ayala J.A., Hess W.M., Kistler F.D., Joyce G.A. (1991). Carbon-black−elastomer interaction. Rubber Chem. Technol..

[18] Demirhan E., Kandemirli F., Kandemirli M. (2007). The effects of furnace carbon blacks on the mechanical and the rheological properties of SBR1502 styrene butadiene rubber. Mater. Des..

[19] Ao G., Hu Q., Kim M.S. (2008). Properties of activated carbon blacks filled SBR rubber composites. Carbon Lett..

[20] Ma J.H., Zhang L.Q., Wu Y.P. (2013). Characterization of filler-rubber interaction, filler network structure, and their effects on viscoelasticity for styrene-butadiene rubber filled with different fillers. J. Macromol. Sci. B: Phys..

[21] Yang H., Gong J., Wen X., Xue J., Chen Q., Jiang Z., Tian N., Tang T. (2015). Effect of carbon black on improving thermal stability, flame retardancy and electrical conductivity of polypropylene/carbon fiber composites. Comp. Sci. Technol..

[22] Zhang Q., Wang J., Yu J., Guo Z.X. (2017). Improved electrical conductivity of TPU/carbon black composites by addition of COPA and selective localization of carbon black at the interface of sea-island structured polymer blends. Soft Matter.

[23] Qu M., Deng F., Kalkhoran S.M., Gouldstone A., Robisson A., Van Vliet K.J. (2011). Nanoscale visualization and multiscale mechanical implications of bound rubber interphases in rubber−carbon black nanocomposites. Soft Matter.

[24] Wolff S., Wang M.J., Tan E.H. (1993). Filler−elastomer interaction. Part VII. Study on bound rubber. Rubber Chem. Technol..

[25] Leblanc J.L. (1997). A molecular explanation for the origin of bound rubber in carbon black filler rubber compounds. J. Appl. Polym. Sci..

[26] Choi S.S. (2000). Characterization of bound rubber of filled styrene-butadiene rubber compounds using pyrolysis-gas chromatography. J. Analyt. Appl. Pyrol..

[27] Choi S.S., Kim I.S. (2002). Filler–polymer interactions in filled polybutadiene compounds. Eur. Polym. J..

[28] Kablov V.F., Patryuk I.P. (2016). The influence of the carbon black morphology on the interphase layer content in filled elastomers. Inter. Polym. Sci. Technol..

[29] Choi S.S., Ko E. (2014). Novel test method to estimate bound rubber formation of silica-filled solution styrene-butadiene rubber compounds. Polym. Test..

[30] Tyczkowski J., Krawczyk I., Woźniak B., Martin-Martinez J.M. (2009). Low-pressure plasma chlorination of styrene–butadiene block copolymer for improved adhesion to polyurethane adhesives. Eur. Polym. J..

[31] Goldstein J.I., Newbury D.E., Michael J.R., Ritchie N.W.M., Scott J.H.J., Joy D.C. (2018). Scanning Electron Microscopy and X-ray Microanalysis.

[32] Tyczkowski J., Kierzkowska-Pawlak H., Sielski J., Krawczyk-Kłys I. (2020). Low-temperature plasma modification of styrene–butadiene block copolymer surfaces for improved adhesion – a kinetic approach. Polymers.

[33] Roy S., Yue C.Y. (2011). Surface modification of COC microfluidic devices: A comparative study of nitrogen plasma treatment and its advantages over argon and oxygen plasma treatments. Plasma Proc. Polym..

[34] The GIMP Development Team GIMP, 2020. https://www.gimp.org.

